# Editorial: Occupational exposure and cardiometabolic disorders

**DOI:** 10.3389/fpubh.2023.1171033

**Published:** 2023-06-16

**Authors:** Kun Qin, Peng Jia, Shujuan Yang

**Affiliations:** ^1^School of Resource and Environmental Sciences, Wuhan University, Wuhan, China; ^2^Hubei Luojia Laboratory, Wuhan, China; ^3^West China School of Public Health and West China Fourth Hospital, Sichuan University, Chengdu, China; ^4^Department of Health Management Center, Clinical Medical College & Affiliated Hospital, Chengdu University, Chengdu, China; ^5^School of Public Health, Wuhan University, Wuhan, China; ^6^International Institute of Spatial Lifecourse Health (ISLE), Wuhan University, Wuhan, China

**Keywords:** cardiometabolic disorder, metabolic syndrome, cardiovascular disease, environmental exposure, occupational exposure, occupational protection

Cardiometabolic disorders are a group of metabolic dysfunctions mainly characterized by central adiposity, dyslipidemia, insulin resistance, impaired glucose tolerance, and hypertension, also referred to as metabolic syndrome (MetS) (1). They could further lead to non-communicable diseases, such as cardiovascular diseases (CVDs) and diabetes, accounting for a large burden of morbidity and mortality worldwide. The prevalence of cardiometabolic disorders has been rising along with urbanization, to a large extent due to the increasing unhealthy lifestyles, such as unbalanced and high-energy diet and sedentary behaviors (2). Moreover, due to the Coronavirus Disease 2019 (COVID-19) pandemic, governments in many countries have implemented rigorous prevention and control measures including lockdown, school closure, working from home, and constraining services of non-essential businesses (e.g., recreational facilities), which have unavoidably affected individual lifestyles and behaviors in health-compromising ways. For example, eating, food ordering, and physical activity patterns have dramatically changed during COVID-19 lockdown, which could all lead to the increased risk for cardiometabolic disorders (3–6).

## Occupational exposure: an increasingly recognized risk factor of cardiometabolic disorders

With approximately a third to half of the day spent at work, occupational exposure has been increasingly considered important determinants of cardiometabolic disorders. Some occupational exposures that may lead to cardiometabolic disorders and CVDs have been studied, such as exposure to physical (e.g., noise, radiation, extreme weather), chemical (e.g., air pollutants, poisonous substance), biological (e.g., microbes, viruses), and psychological factors (e.g., stress). Some feasible ways of counteracting negative health effects of those exposures for preventing cardiometabolic disorders have been found, such as earmuff and mask wearing, protective clothing, and psychological consultation. However, the existing findings have been inconsistent and there are more occupation-related factors of cardiometabolic disorders to be investigated, such as physiological factors (e.g., circadian rhythm disruption), psychological factors (e.g., depression), and other lifestyle-related factors (e.g., sedentary behaviors). Also, effectiveness and efficiency of the currently used preventive measures for those factors have not been fully assessed. Given the complex relationships among occupational exposure, preventive measures, and cardiometabolic disorder risks, plus the emerging occupations and changing features of traditional occupations, it is worthwhile to keep and even enhance investments on examining the effects of occupational exposure and the effectiveness of protection strategies.

## New evidence from observational studies

In epidemiological studies, MetS is usually defined as the presence of at least three of the five components, i.e., central obesity, high blood pressure, high triglycerides, low high-density lipoprotein cholesterol (HDL-c), and hyperglycemia. A level of metabolic risk is measured by the number of MetS components one presents at the time of the survey. Most of the observational studies examining the prevalence and risk factors of MetS have been conducted in western countries before (7). Recently, an increasing number of MetS studies on the basis of large population cohorts in eastern countries have emerged (1, 8). For example, a recent retrospective cohort study of the 3,777 employees (aged 44.9 ± 12.9 years on average, with 42.2% being females) from five institutions in Luzhou, China, receiving an annual physical examination during 2018–2020, shows that the prevalence of MetS and the level of metabolic risk had significantly increased in general after the COVID-19 lockdown (Xu et al.). This may be caused by the unhealthy lifestyles during COVID-19 lockdown, such as life rhythm disruption. Such trends in occupational populations appealed targeted measures, e.g., health education, to remedy post-pandemic public health issues.

Another study of the 92,869 subjects without CVD or cancers on the basis of the China Cardiometabolic Disease and Cancer Cohort, a nationwide prospective cohort recruiting 193,846 participants during 2011–2012 (baseline) and following them up during 2014–2016, has found that depression, an important psychological risk factor of MetS, may interact with MetS to affect the risk for major CVD events (Chen et al.). Moreover, the higher level of metabolic risks may intensify the association between depression and the increased CVD risk. This association may also be amplified by the higher level of occupation-related lifestyle risk, measured by the number of unhealthy behaviors (i.e., unhealthy diet, physical inactivity, non-ideal alcohol intake, and current smoking). Given the synergistic effects of multiple occupational risk factors, this study suggested simultaneous management on those risk factors.

## New evidence from randomized clinical trials

Considering severe consequences of adverse behaviors in existing observational studies, there are some new efforts targeting unhealthy lifestyle rectification to promote effective occupational protection and improve cardiometabolic health. A 12-month randomized controlled trial (RCT) in Finland was conducted to examine the effects of a physical activity intervention on body composition and cardiometabolic health indicators (Leskinen et al.). The 231 elders retiring from the public sector between January 2016 and December 2018, with a mean age of 65.2 ± 1.1 years and 83% being females, participated in the RCT baseline after their retirement for about 1 year. They were randomly divided into intervention and control groups, with the intervention group assigned to utilize a commercial activity tracker to boost physical activity with a daily goal. Body composition and cardiometabolic health indicators, including body weight, fat mass, fat free mass, waist circumference, blood pressure (systolic and diastolic), glycated hemoglobin (HbA1c), fasting plasma glucose, triglycerides, HDL-c, low-density lipoprotein cholesterol (LDL-c), and high-sensitivity C-reactive protein (hs-CRP), all presented no differences in the changes between groups over 12 months except blood pressure.

Another 12-week RCT in Southwest Germany was conducted during COVID-19 to investigate the effects of regular attendance in a 12-week web-based weight loss program on cardiometabolic risk factors and one's risk for complications of COVID-19 infection (Brame et al.). Through print and online media, the 92 adults with overweight/obesity, with a mean age of 50.0 ± 10.8 years, a mean weight of 30.5 ± 2.1 kg/m^2^, and 76.1% being females, were recruited between January and August 2020 and separated into two groups randomly. Cardiometabolic risk factors included body weight, body mass index (BMI), waist circumference, blood pressure (systolic and diastolic), flow-mediated dilatation, HbA1c, triglycerides, HDL-c, and LDL-c. A construct named *COVIDAge* was newly developed by combining chronological age and different lifestyle factors associated with an increased risk for COVID-19, to estimate one's risk for complications of COVID-19 infection, with a higher *COVIDAge* indicating a higher risk of COVID-19. The intervention group showed significantly greater reductions in the three anthropometric outcomes, systolic blood pressure, and *COVIDAge* than the control group. It implies that web-based health interventions on facilitating lifestyle changes may also be effective for accessing personalized health guidance and thus alleviating the increasing occupation-related cardiometabolic risk.

## Outlook for future studies

The recent studies have further consolidated the associations between occupational exposure and cardiometabolic disorders and implemented prevention strategies. The effects of occupational exposure on cardiometabolic disorders may be closely associated with unhealthy lifestyles and become particularly prominent during public health emergencies, such as COVID-19. It is of great importance to obviate occupational exposure and maintain cardiometabolic health of occupational populations.

Some efforts are on the way. For example, an ongoing pilot study recruited the 44 participants, clustered in two groups, from a university and a council in the east of England region, UK (Ojo et al.). It integrated the BCW (Behavior Change Wheel) and APEASE (Acceptability, Practicability, Effectiveness/cost-effectiveness, Affordability, Safety/side-effects, Equity) criteria to evaluate occupational exposures at workplace and identify intervention functions, policy categories, and behavior change techniques that were most appropriate or have the highest possibility of success in delivering the desired change. These interventions on promoting lifestyle changes may associate with cardiometabolic health and further affect CVD risks indirectly. The protocol highlighted the feasibility of a multi-component intervention in alleviating occupational exposures (e.g., sedentary behaviors) and informed the effective design and implementation of occupational interventions through promoting positive lifestyle behaviors.

Multi-pronged occupational protection strategies and healthy lifestyle-promoting policies are still warranted. Future studies are suggested to focus on evaluating the burden of various specific occupational exposures on cardiometabolic disorders and reveal underlying mechanisms, including the further interaction and potential spillover effects between lifestyle-related occupational exposure and cardiometabolic disorders. Also, due to multi-dimensionality and complexity of occupational risk factors of cardiometabolic disorders, advanced methods and transdisciplinary collaboration are necessary to design and implement novel research in the future (9).

## Author contributions

SY and PJ conceived the idea. KQ drafted the manuscript. All authors revised the manuscript and approved the final version.
